# Anti-Quorum Sensing and Anti-Biofilm Activity of *Pelargonium × hortorum* Root Extract against *Pseudomonas aeruginosa*: Combinatorial Effect of Catechin and Gallic Acid

**DOI:** 10.3390/molecules27227841

**Published:** 2022-11-14

**Authors:** Fatma M. Abdel Bar, Manal A. Alossaimi, Engy Elekhnawy, May Abdullah Abulrahman Alzeer, Amal Abo Kamer, Ehssan Moglad, Mai H. ElNaggar

**Affiliations:** 1 Department of Pharmacognosy, College of Pharmacy, Prince Sattam bin Abdulaziz University, Al-Kharj 11942, Saudi Arabia; 2 Department of Pharmacognosy, Faculty of Pharmacy, Mansoura University, Mansoura 35516, Egypt; 3 Department of Pharmaceutical Chemistry, College of Pharmacy, Prince Sattam bin Abdulaziz University, Al-Kharj 11942, Saudi Arabia; 4 Pharmaceutical Microbiology Department, Faculty of Pharmacy, Tanta University, Tanta 31527, Egypt; 5 Department of Pharmaceutics, College of Pharmacy, Prince Sattam bin Abdulaziz University, Al-Kharj 11942, Saudi Arabia; 6 Department of Pharmacognosy, Faculty of Pharmacy, Kafrelsheikh University, Kafrelsheikh 33516, Egypt

**Keywords:** *Pelargonium*, Geraniaceae, simultaneous docking, gallic acid, catechin, quorum sensing, bacterial pathogenicity

## Abstract

HPLC-UV was used to compare the major constituents of two *Pelargonium × hortorum* cultivars and *Pelargonium sidoides* root extract. It revealed the presence of catechin and gallic acid in high concentrations and the absence of umckalin in *P. × hortorum* root extracts. The antibacterial activity of these extracts was screened against 19 *Pseudomonas aeruginosa* clinical isolates. *P. × hortorum* root extracts showed the lowest MIC values (512–1024 µg/mL). This activity was concluded to be attributable to the high concentrations of catechin and gallic acid. The anti-biofilm activity of catechin, gallic acid, and their combination was examined by a crystal violet assay. The combination reduced the percentage of strong and moderate biofilm-forming isolates from 52.63% to 5.26%. The impact on *las*I and *las*R genes expression using qRT-PCR and simultaneous docking against *Las*R protein was explored. The combination downregulated *las*I and *las*R gene expression in eight and six *P. aeruginosa* isolates, respectively, and showed the greatest docking score. Additionally, the in vivo protection capability of this combination in infected mice showed enhancement in the survival rate. Our study revealed the potential biofilm and quorum-sensing-inhibitory activity of the catechin and gallic acid combination as a novel alternative to inhibit bacterial pathogenicity.

## 1. Introduction

*Pelargonium × hortorum*, commonly known as zonal or garden geranium, is one of the most popular ornamental crops that is believed to be originated by cross hybridization between several wild species of genus *Pelargonium* section Ciconium [1,2]. It is an economically important taxon of the family Geraniaceae due to its value in the florist industry worldwide [2].

*Pelargonium sidoides* (Umckaloabo) is a medicinal plant belonging to the genus *Pelargonium*. It is used traditionally for the treatment of many disorders, including upper respiratory tract infections, such as bronchitis, common cold, and sinusitis [3]. The main constituents of *P. sidoides* showed antibacterial and antiviral activities against several respiratory pathogens [4,5,6]. Despite the popularity of *Pelargonium × hortorum* plants, and the medicinal importance of other species belonging to the same genus, the research studies investigating their phytochemical composition and medicinal uses are limited. Previous phytochemical research indicated that the therapeutic effect of *P. sidoides* is partly attributed to catechin, gallic acid, and highly oxygenated coumarins, including scopoletin, and umckalin [7,8]. Therefore, the current study was aimed at tracing the presence of these four biological and chemotaxonomic markers in different parts of two *Pelargonium × hortorum* cultivars grown in Saudi Arabia as ornamental flowering plants and comparing the obtained results to that of the root extract of the medicinal plant, *P. sidoides*, using the HPLC-UV technique.

Disseminating antibiotic resistance among the pathogenic bacterial isolates is regarded as a major public health concern owing to the high number of patients suffering from untreatable serious infections [9]. *Pseudomonas aeruginosa* is a ubiquitous opportunistic Gram-negative pathogenic bacterium. It is one of the major causes of hospital-acquired infections [10,11]. It can cause a wide range of antibiotic-resistant infections that are accompanied by high rates of morbidity and mortality [11,12]. Bacteria have various virulence factors such as biofilms which increase the bacterial ability to develop resistance to antibiotics by hindering their diffusion through the biofilm matrix [13]. In addition, biofilms can mediate the horizontal transfer of antibiotic resistance genes among bacterial cells impeded in biofilms [14,15]. Virulence quenching is an important strategy in the battle against the infections caused by multidrug-resistant bacteria [13]. This approach focuses on inhibiting bacterial quorum sensing, decreasing the bacterial pathogenesis, and enhancing the immune system of the host to destroy such pathogenic bacteria. This approach has an important advantage as it does not harm the host normal flora [16]. 

*Las*I/*Las*R is a major pathway used by *P. aeruginosa* for the regulation of quorum sensing using autoinducers such as acyl-homoserine lactones which activate the transcriptional regulators in the bacterial cells and induce gene transcription. Therefore, the inhibition of quorum sensing (QS) by targeting *Las*I and *Las*R enzymes could help in decreasing the antibiotic resistance and pathogenicity of *P. aeruginosa* [17,18]. 

In this study, the antibacterial activity of the methanolic extracts of different parts of *Pelargonium × hortorum* cultivars and their major biological markers against *P. aeruginosa* clinical isolates was investigated. We also elucidated the anti-biofilm and anti-quorum-sensing potential of the tested biological markers. Moreover, their mechanism of action was investigated using qRT-PCR and *in silico* docking studies against the main targets involved in the coordination of QS in *P. aeruginosa.*

## 2. Results and Discussion

### 2.1. Chromatographic Analysis

**Figure 1:**
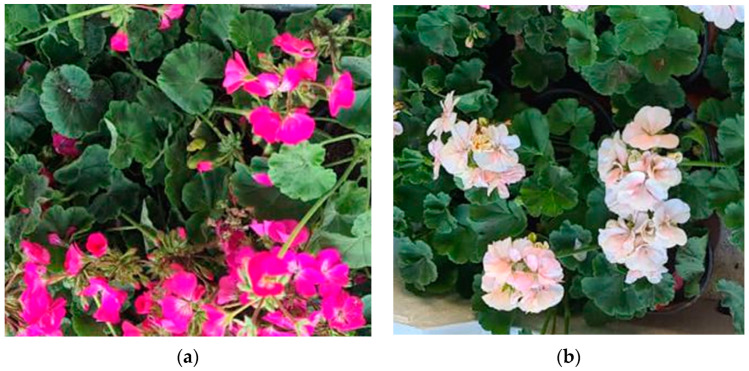
*Pelargonium × hortorum* (PH) cultivars; (**a**) with pink flowers (PPH); (**b**) with white-to-rose flowers (WPH).

**Figure 2:**
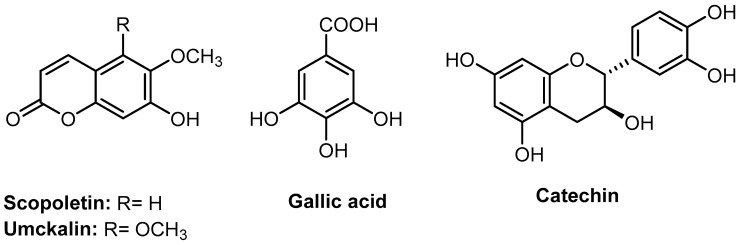
Analytical standards used in the phytochemical distinction between *Pelargonium × hortorum* cultivars and *Pelargonium sidoides*.

*Pelargonium × hortorum* and *P. sidoides* are important plants belonging to the same genus in the family Geraniaceae. However, there is no established analytical method that could compare the phytochemical profile of both plants. In this study, two *P. × hortorum* cultivars, with pink flowers (PPH), Figure 1a, and white-to-rose flowers (WPH), Figure 1b, were collected. The different parts of the collected plants were separated into roots, flowers, leaves, and stems. On the other hand, *P. sidoides* root extract was obtained from the commercial kalobin^®^ solution. The method used for the comparative study is based on the presence of four reported biomarkers, including catechin, gallic acid, scopoletin, and umckalin (Figure 2) in the root extract of *P. sidoides* [6,19]. 

The conditions of the simultaneous chromatographic separation were optimized by changing one parameter while maintaining other parameters constant at a time. These parameters included the mobile phase, stationary phase, wavelength, and samples preparation method. Several trials were performed with separate values of acetonitrile-water ratio and pH. The most appropriate chromatographic settings for the current study were using the acetonitrile-water ratio, 20:80 *v/v* (pH 3.0), a flow rate of 1 mL/min, an injection volume of 20 µL, a run time of 25 min, on a Waters XBridge^®^ C_18_ column (200 mm L. × 4.6 mm W., and a particle size of 5 µm) employed at 30 °C and a wavelength of 210 nm. This analytical method is fully validated in accordance with the guidelines of the International Council for Harmonization (ICH) to show that it is linear, accurate, precise, and fast. 

**Figure 3:**
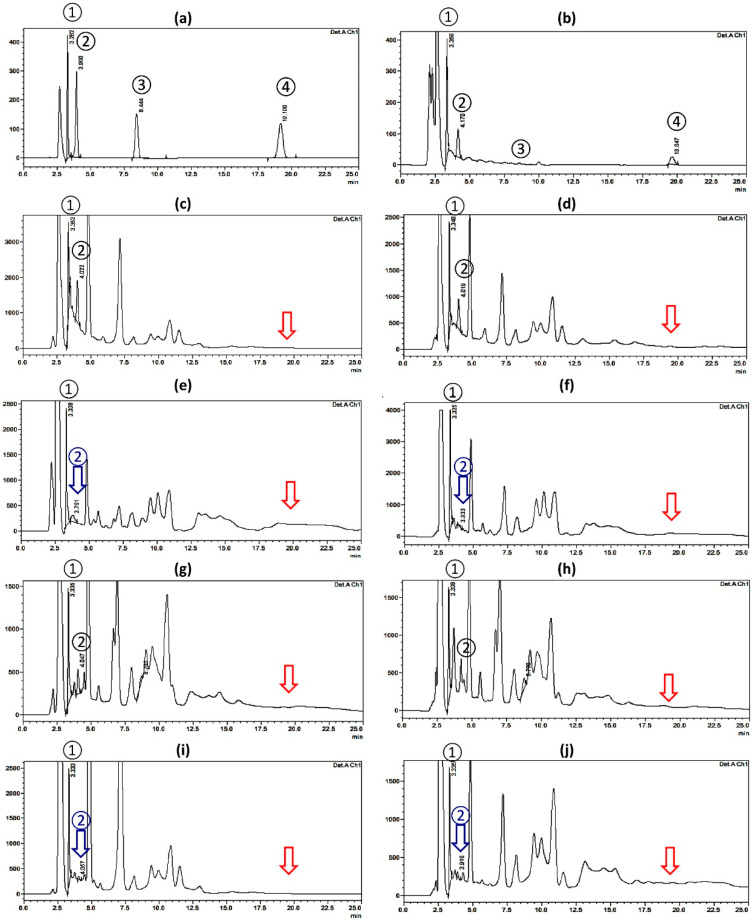
HPLC-UV chromatograms (at 210 nm) on a RP-C_18_ column with reference standards peaks; (**1**) Gallic acid, (**2**) Catechin, (**3**) Scoploletin, and (**4**) Umckalin. (**a**) Mixture of reference standards biomolecules; and different plant extracts at a concentration of 10 mg/mL of; (**b**) *Pelargonium sidoides* root extract, (**c**) *Pelargonium × hortorum* with pink flowers (PPH) root, (**d**) *Pelargonium × hortorum* with white flowers (WPH) root, (**e**) PPH flower, (**f**) WPH flower, (**g**) PPH leaf, (**h**) WPH leaf, (**i**) PPH stem, and (**j**) WPH stem. The blue arrows refer to undetected catechin peaks and the red arrows refer to undetected umckalin peaks.

The four investigated biomolecules were simultaneously eluted yielding symmetrical peaks within 25 min as a minimum time of analysis, Figure 3. The retention time values were 3.3, 4.0, 8.4, and 19.2 min, for gallic acid, catechin, scopoletin, and umckalin, respectively (Figure 3a). 

The HPLC-UV chromatogram of *P. sidoides* showed the presence of detectable peaks corresponding to the investigated biomolecules with concentrations of 22, 10.96, 0, and 3.09 μg/mL for gallic acid, catechin, scopoletin, and umckalin, respectively (Table 1 and Figure 3b). 

The root extracts of PPH and WPH showed the presence of appreciable amounts of gallic acid (163.69 and 98.75 μg/mL, respectively) and catechin (172.56 and 104.59 μg/mL, respectively) with observable higher concentrations in PPH as displayed in Figure 3c,d, and Table 1. The presence of large amounts of catechin in the roots of PPH and WPH was further confirmed by TLC against the reference sample that showed an orange spot (R*_f_* 0.11) on silica gel GF_254_, using the solvent system; CHCl_3_-CH_3_OH, 90:10 *v*/*v* (Figure S1). Similarly, the PPH and WPH leaf extracts showed the presence of gallic acid (92.29 and 82.62 μg/mL) and catechin (44.10 and 47.98 μg/mL), respectively, but in much lower concentrations than that of the root extracts. The flower and the stem extracts of both cultivars showed the absence of clear peaks for catechin. However, they showed the presence of major peaks for gallic acid in relatively high concentrations in PPH and WPH flower extracts (124.99 and 213.50 μg/mL, respectively) than in stem extracts (133.96 and 85.11 in PPH and WPH, respectively). Regarding scopoletin, although ambiguous peaks at R*_t_* range from 8.2 to 8.6 min were detected in different extracts of *P. × hortorum* cultivars, TLC chromatograms against the reference standard revealed the absence of fluorescent peaks at UV_254_ and UV_336_ lights corresponding to this compound confirming its absence from these plants; Figure S1a,b (See Supplementary Materials**)**. Moreover, none of the investigated *P. × hortorum* extracts showed the presence of peaks corresponding to the coumarin biomarker, umckalin; Figure 3c–j.

Therefore, by comparing the chromatograms (Figure 3) and the detected concentrations of the studied biomolecules (Table 1), it could be clearly concluded that the extracts of *P. × hortorum* cultivars can be distinguished from that of the medicinally used *P. sidoides* species by the absence of the prominent characteristic peak for umckalin. This result is in full agreement with the conclusion drawn by Harborne and Williams [19] which indicated that umckalin alone is sufficient to be used as a chemotaxonomic marker to distinguish *P. sidoides* from garden geraniums. We can also conclude the presence of catechin and gallic acid combination in higher concentrations in *P. × hortorum* root extracts in comparison to *P. sidoides* root extract. The root extract of *Pelargonium × hortorum,* especially the cultivar with the pink flowers (PPH), can be considered as a rich source for catechin and gallic acid phytochemical combination.

Phenolic compounds, such as flavonoids, anthocyanins, coumarins, and hydroxycinnamic acids, are biosynthesized in plants via the shikimate pathway [20,21]. Phenylalanine ammonia-lyase (PAL) is a key enzyme involved in this biosynthetic pathway where it catalyzes the non-oxidative deamination of L-phenylalanine to trans-cinnamic acid and ammonium [20,21]. Several studies revealed that the difference in the protein expression of PAL is related to the level of biosynthesis of phenolic compounds in plants [22,23]. The two studied *P. × hortorum* plants are cultivated varieties “cultivars” that refer to man-made variations within the plant species to obtain desirable characteristics. Creating new cultivars with superior traits is crucial, especially in the field of growing medicinal plants [24]. In this study, the cultivar with pink flowers (PPH) showed higher concentrations of both gallic acid and catechin. This may be attributable to the high expression of the genes responsible for PAL protein biosynthesis in this cultivar.

### 2.2. Biological Activity

#### 2.2.1. Antibacterial Activity

*P. aeruginosa* can cause severe life-threatening infections, particularly in patients who are suffering from a weak immune system such as AIDS and cancer patients. Unfortunately, many isolates of *P. aeruginosa* can form biofilm which enables them to be much more resistant to the available antibiotics [25]. Consequently, it is essential to discover novel alternatives to the currently used antibiotics. Plants are a rich source for various bioactive agents which possess a wide range of biological potentials. Here, we explored the antibacterial activity of the methanolic extracts of different parts of *Pelargonium × hortorum* cultivars and *P. sidoides* root extract against *P. aeruginosa* isolates using the agar well diffusion method. Interestingly, all of the tested plant extracts exhibited antibacterial activity as they resulted in the appearance of inhibition zones around the wells. Thus, their MIC values were detected using the broth dilution method. Among the tested extracts, the root extracts of PPH and WPH showed the most promising antibacterial activity against the tested *P. aeruginosa* isolates. Noticeably, the root extract of PPH showed better results than that of WPH. The PPH root extract exhibited minimum inhibitory concentration (MIC) values ranging from 512 to 1024 µg/mL against 18 isolates, while WPH root extract exhibited MIC values within the same range against only 14 isolates, Table S1 (See Supplementary Materials). Other plant extracts including *P. sidoides* root extract exhibited higher MIC values of ≥2048 µg/mL against most of the tested isolates.

Catechin and gallic acid are polyphenolic compounds that are widely distributed in numerous foods and plants and they are renowned for their various biological activities [26,27]. Both biomolecules have been reported to exhibit antimicrobial activity against several bacterial species [28,29,30]. Therefore, it was concluded that the antibacterial activity of the most active PPH root extract could be attributed to the presence of catechin and gallic acid in high concentration as revealed by the HPLC-UV analytical study.

The biological activity of the herbal plant extracts is usually attributed to the presence of combination of multiple compounds [31]. Hence, the antibacterial activity of the two biomolecules, catechin and gallic acid, individually or in combination, were also tested. Remarkably, both catechin and gallic acid exhibited better antibacterial activity with lower MIC values ranging from 64 to 1024 µg/mL as depicted in Table S2 (See Supplementary Materials). In order to elucidate the relevance of the antibacterial activity of catechin and gallic acid, we compared their MICs to the MICs of the standard antibiotic (ciprofloxacin). Remarkably, their MICs were relatively close to the MICs of ciprofloxacin which had MIC values ranging from 16 to 256 µg/mL as shown in Table S2 (See Supplementary Materials). Additionally, their combination exhibited a synergistic activity as they demonstrated fractional inhibitory concentration index (FICI) value lower than 0.5. The FICI lower than one means synergy, as in this case, less concentration of the tested drugs would be required for production of the same effect as the drugs alone [32]. Therefore, both biomolecules are recommended to be used in a combination therapy which can result in reducing the ability of bacteria to develop resistance and decreasing the dose and the adverse effects of each compound in the combination [33].

#### 2.2.2. Anti-Biofilm and Anti-Quorum-Sensing Activity

Biofilm represents an attractive target for the anti-virulence compounds because biofilm eradication can produce a negative impact on the progression of infections [34]. Hence, it is considered a promising strategy for preventing multidrug-resistant bacteria. Therefore, we explored the anti-biofilm potential of catechin, gallic acid, and their combination using a crystal violet assay. Previous studies explored the anti-quorum sensing and anti-biofilm activities of catechin or gallic acid alone [35,36,37]. However, to our knowledge, this is the first time to explore the activity of their combination. Interestingly, the combination of catechin and gallic acid exhibited more potent anti-biofilm activity in comparison to the individually tested molecules. The combination exhibited a remarkable anti-biofilm activity as it reduced the percentage of the strong and moderate biofilm-forming isolates from 52.63% to 5.26% as revealed in Table 2.

**Figure 4:**
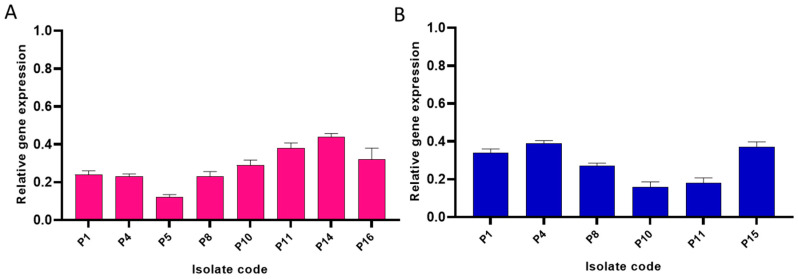
Bar chart showing downregulation of the expression of (**A**) *las*I and (**B**) *las*R genes. In the tested isolates by combinatorial treatment with catechin and gallic acid.

During the biofilm formation, bacterial cells communicate with each other via the QS system which induces biofilm formation, regulates the metabolic activity of the planktonic cells, and increases their virulence. *Las*I/*Las*R is a major pathway used by *P. aeruginosa* for the regulation of quorum sensing using autoinducers such as acyl-homoserine lactones which activate the transcriptional regulators in the bacterial cells and induce gene transcription. As the combination of catechin and gallic acid revealed the best anti-biofilm activity, we tested its impact on the gene expression of the QS genes (*las*I and *las*R) in ten *P. aeruginosa* isolates which had a strong and moderate biofilm formation ability before treatment using qRT-PCR. Remarkably, the combinatorial treatment revealed a significant downregulation in *las*I and *las*R gene expression in eight and six *P. aeruginosa* isolates, respectively, as revealed in Figure 4.

#### 2.2.3. In Vivo Assay

**Figure 5:**
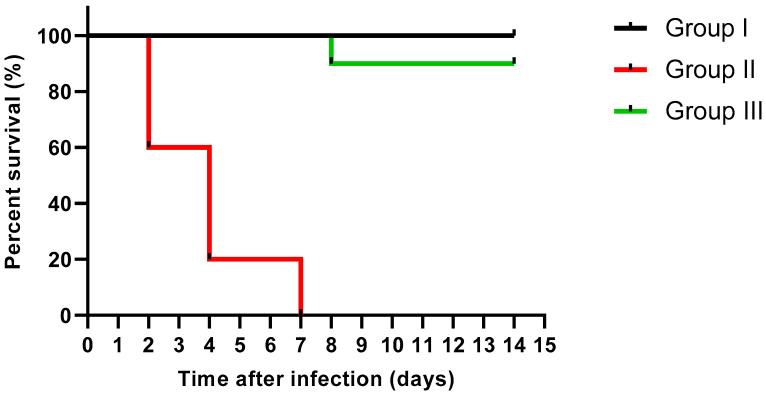
Kaplan–Meier survival curve of the experimental groups after treatment with catechin and gallic acid combination.

Moreover, the protective effect of catechin and gallic acid combination was investigated in a mice model to evaluate its *in vivo* antibacterial activity. The survival curve of the experimental groups was constructed as revealed in Figure 5. There was no death among the non-infected normal mice (group I). Regarding group II, representing the mice infected with untreated *P. aeruginosa*, four mice died after two days, four mice died after four days, and the rest died after one week. Interestingly, one mouse only died after eight days in group III representing the mice infected with *P. aeruginosa* treated with gallic acid and catechin combination and the remaining mice remained alive till the end of the experiment. The combination revealed a significant (*p* < 0.05) decline in the killing capability of the treated isolates when compared to the non-treated ones.

### 2.3. Docking Study

**Figure 6:**
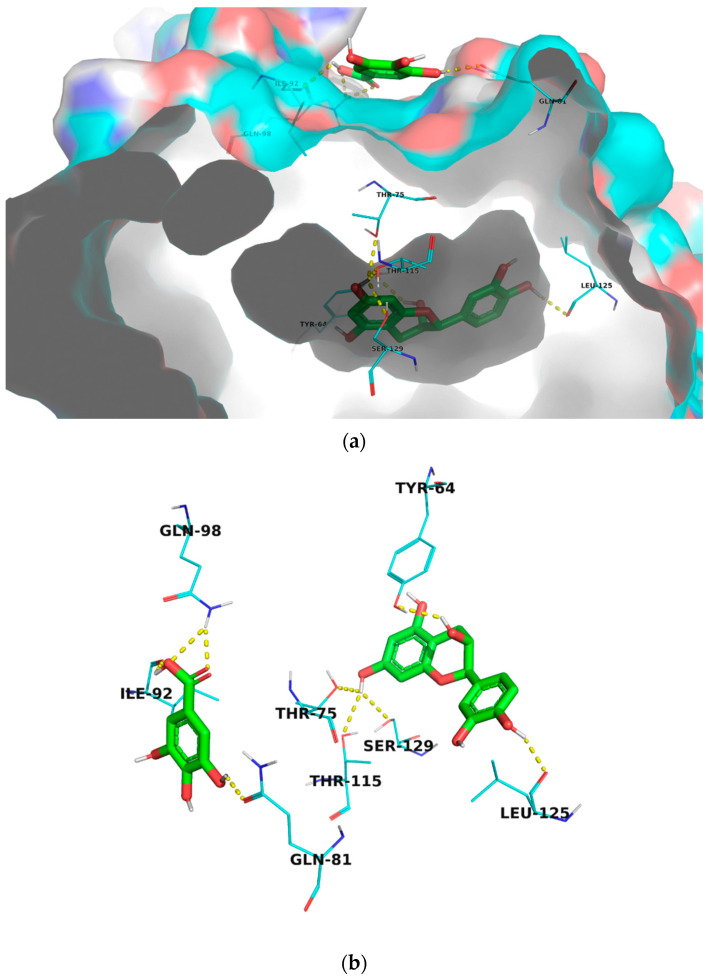
Molecular model binding of simultaneously docked catechin and gallic acid to the transcriptional activator receptor (*Las*R, PDB code: 2UVO); (**a**) *Las*R protein in surface presentation demonstrating the docking sites of catechin and gallic acid (**b**) Line presentation of *Las*R amino acid residues involved in interaction with catechin and gallic acid.

In this article, we performed a molecular docking study against the transcriptional activator receptor protein (*Las*R) that is essential for QS in *P. aeruginosa* to investigate the mechanism of action of gallic acid, catechin, and their combination. Simultaneous docking is a new function that has been implemented into the new version of AutoDock Vina 1.2.0 which enables the docking of multiple ligands to the same target simultaneously [38]. We have used this functionality for the first time to dock catechin and gallic acid to the active site of *Las*R simultaneously. Interestingly, the docked combination showed the highest docking score (−13.07 kcal/mol, Table 3) which is greater than the individually tested molecules and the co-crystallized autoinducer, 3-oxo-C12-acylhomoserine lactone. Visualization of the molecular model of binding showed that catechin was docked into the ligand-binding domain (LBD) of *Las*R and formed hydrogen bonds with Thr-115, and Ser-129 amino acid residues that are reported to be involved in the interaction with the autoinducer ligand [39]. It also showed hydrogen bonding interaction with Tyr-64, Thr-75, and Leu-125 amino acid residues in the LBD (Figure 6). While gallic acid was highly fitted into a different pocket close to the LBD and formed hydrogen bonds with Gln-81, Ile-92, and Gln-98 amino acid residues (Figure 6) that may be involved in the dimerization of *Las*R protein. The obtained results support the high potential of catechin and gallic acid combination in inhibiting the *Las*R enzyme and interrupting QS and biofilm formation in *P. aeruginosa.*

## 3. Material and Methods

### 3.1. Plant Material

Two *Pelargonium × hortorum* cultivars were selected for this study; one with pink flowers (PPH), Figure 1a, and the other with white-to-rose flowers (WPH), Figure 1b. The identity of these plants was confirmed by Prof. Ibrahim Mashaly, Professor of Plant Ecology, Faculty of Science, Mansoura University, Egypt. The different parts of the plants were separated into leaves, flowers, stems, and roots to be used in the comparative HPLC-UV analytical study against *P. sidoides*.

### 3.2. Extraction

The different dried powdered parts of WPH, including 15 g of leaves, 1.9 g of flowers, 3.5 g of stems, and 3.5 g of roots, in addition to 12 g of leaves, 3 g of flowers, 6 g of roots, and 9.5 g of stems of PPH were extracted separately by rinsing with CH_3_OH (3 × 100 mL). The CH_3_OH extracts were combined and concentrated using a rotary evaporator at 45 °C then allowed to dry at room temperature. The dried total CH_3_OH extracts of WPH yielded leaf extract (2730.2 mg, 18.2% *w*/*w*), flower extract (309.2 mg, 16.27% *w*/*w*), root extract (468.6 mg, 13.38% *w*/*w*), and stem extract (704.2 mg, 20.12% *w*/*w*). While the dried total CH_3_OH extracts of PPH yielded leaf extract (2360.3 gm, 19.66% *w*/*w*), flower (308.2 mg, 10.27% *w*/*w*), root extract (798.9, 13.31% gm *w*/*w*), and stem extract (1363.9, 14.35% gm *w*/*w*). The obtained dry extracts were kept in closed containers in a refrigerator to be used in HPLC analyses.

### 3.3. Chromatographic Analysis

#### 3.3.1. Mobile Phases

Acetonitrile, CH_3_CN (HPLC grade, ≥99.9%, SIGMA-ALDRICH^®^, St. Louis, MO, USA), methanol, CH_3_OH (HPLC grade, ≥99.9%, Fisher Chemical, Waltham, MA, USA), orthophosphoric acid, H_3_PO_4_ (HPLC, 85–90%, FLUKA^®^, Seelze, Germany) were used in the preparation of the mobile phase. Nylon membrane filters of 0.45 μm pore size were used in solvent filtration (HNWP, MERCK MILLIPORE^®^ Ltd., Billerica, MA, USA). Ultra-pure (HPLC grade) water was obtained using a Milli-Q water purification system.

#### 3.3.2. Instrument Panel

HPLC chromatographic investigation was achieved utilizing a UFLC-SHIMADZU 1200 series UFLC system (Santa Clara, CA, USA) provided with a binary pump, an autosampler, and an online degasser. Separation was achieved on a C_18_ column, 200 mm L. × 4.6 mm W., and 5 µm particle size (Waters XBridge^®^, Waters, Ireland). The system was supplied with an HPLC photodiode-array detector (SPD-20 A) set at a wavelength of 210 nm. The HPLC instrument was interfaced with a computer installed with LabSolutions software on a Microsoft Windows 7.0 operating system.

#### 3.3.3. Chromatographic Conditions

Isocratic elution was applied using a mobile phase composed of CH_3_CN-H_2_O (25:75, *v*/*v*) with an adjusted pH at 3.0 by adding drops of 10% H_3_PO_4_. The mobile phase was then filtered by a 0.45 µm membrane filter and degassed by a sonicator for 15 min. The column was set at 30 °C as the operating temperature. An injection volume of 20 µL, a flow rate of 1 mL/min, and a total run time of 25 min were maintained during investigations.

#### 3.3.4. Analytical Standards

Four standards were used in this study, including umckalin (HPLC purity ≥ 95.0%, CAS #: 43053-62-9, SIGMA-ALDRICH), scopoletin (HPLC purity ≥ 97.0%, CAS #: 92-61-5, SIGMA-ALDRICH), (+)-catechin (HPLC purity ≥ 99.0%, CAS #: 154-23-4, SIGMA-ALDRICH), and gallic acid (HPLC purity ≥ 99.0%, CAS Number: 149-91-7, SIGMA-ALDRICH), Figure 2.

#### 3.3.5. Method Development

##### Preparation of Standard Stock Solutions and Calibration Curves

The standard stock solutions of catechin, gallic acid, scopoletin, and umckalin were prepared by dissolving 10 mg of each reference standard in 10 mL of CH_3_OH to obtain 1 mg/mL solution. Then, each solution was further diluted with CH_3_OH to obtain a 100 µg/mL final concentration. To prepare a serial dilution, the obtained stock solution, in each case, was further diluted with CH_3_OH to obtain different concentrations ranging from 0.1 to 1 µg/mL.

##### Preparation of Standard Reference Solution

The working standard solution was obtained by mixing 5 mL of each standard stock solution in a 25 mL volumetric flask, adjusting the volume with CH_3_OH to obtain 200 µg/mL concentration, followed by transferring 8.75 mL of the last solution to a 25 mL volumetric flask and adjusting the volume with (CH_3_CN:CH_3_OH, 1:1 *v*/*v*) to obtain 70 µg/mL concentration. Aliquots of 20 µL of this working solution were injected, the chromatograms were recorded, and the responses of the peak areas were calculated. Percentages of catechin, gallic acid, scopoletin, and umckalin in different parts of the investigated plants were recorded; then, means of the % content were calculated.

##### Preparation of Test Solutions

Different extracts of *Pelargonium × hortorum* cultivars (100 mg) were dissolved in CH_3_OH in a 10 mL volumetric flask, sonicated, filtered through a 0.45 µm membrane filter, and the first few mL of the filtrate was discarded to afford a 10 mg/mL solution. Aliquots of 20 µL of sample solutions were injected in triplicate and the peak areas were measured.

##### Preparations of *Pelargonium sidoides* Test Solution

For HPLC analysis, 1.1 mL of Kalobin^®^ solution, which approximately contains 100 mg of *P. sidoides* dry root extract, were diluted with methanol in a 10 mL volumetric flask, sonicated, and filtered through a 0.45 µm membrane filter to prepare a 10 mg/mL solution. Aliquots of 20 µL of sample solution were injected in triplicate and the peak areas were measured.

### 3.4. In Vitro Antibacterial Activity

#### 3.4.1. Bacteria

Nineteen *P. aeruginosa* clinical isolates were acquired from the culture collection of the Department of Pharmaceutical Microbiology, Faculty of Pharmacy, Tanta University, Egypt. *Pseudomonas aeruginosa* ATCC 27853 was the reference isolate.

#### 3.4.2. Susceptibility Testing

The agar well diffusion method was utilized for testing the susceptibility of *P. aeruginosa* isolates to the tested agents [13]. The plates containing Muller–Hinton agar (MHA) were inoculated with the bacterial suspensions; then, wells were made and filled with the tested agents, ciprofloxacin as a positive control, and 10% dimethyl sulfoxide (DMSO) as a negative control. The plates were incubated overnight at 37 °C and they were inspected for appearance of the inhibition zones around the wells which indicates possessing antibacterial activity. Then, the MIC values were detected by a broth microdilution assay in 96-well microtitration plates as previously described using the previously mentioned positive and negative controls [40]. In order to determine the potential synergy between catechin and gallic acid, the chequerboard assay was used as previously reported [41]. We calculated the FIC according to the following formula:

(1) $$\mathrm{FIC}=\frac{\mathrm{Concentration}\;\mathrm{of}\;\mathrm{catechin}\;\mathrm{or}\;\mathrm{gallic}\;\mathrm{acid}\;\mathrm{in}\;\mathrm{combination}}{\mathrm{MIC}\;\mathrm{of}\;\mathrm{catechin}\;\mathrm{or}\;\mathrm{gallic}\;\mathrm{acid}\;\mathrm{alone}\;}$$

The FICI of catechin and gallic acid in the combination was calculated according to the following formula:

(2) FICI=FIC of catechin+FIC of gallic acid

The two compounds were considered to have a synergetic interaction in the combination if the FICI was equal to or less than 0.5.

#### 3.4.3. Anti-Biofilm Activity

*P. aeruginosa* isolates were screened for their ability to form biofilm by a crystal violet assay as previously described [42,43] via determining the optical density (OD) at 590 nm using ELISA reader (Sunrise Tecan, Austria). The cut-off value (ODc) was calculated. It is defined as three standard deviations (SD) above the mean OD of the negative control. Then, the OD of each isolate was compared to the ODc to determine its biofilm-forming ability. The isolates were grouped into four classes (non-biofilm-forming, weak, moderate, and strong). Then, they were treated with catechin and gallic acid alone and in combination at sub-MIC values (1/4× MIC) and their biofilm-forming ability was explored again.

#### 3.4.4. Anti-Quorum-Sensing Activity by Quantitative Real-Time PCR (qRT-PCR)

The impact of the sub-inhibitory concentration of gallic acid and catechin combination on the QS genes (*las*I and *las*R) [44] was investigated by qRT-PCR using *opr*L gene as a housekeeping gene [41]. Overnight cultures of *P. aeruginosa* (1.0 McFarland) were added to fresh 5 mL Luria–Bertani (LB) broth containing the tested compounds and grown at 37 °C for eight hours with shaking. Then, total RNA was extracted by GeneJET RNA kit (Thermo Scientific, Waltham, MA, USA) and it was converted to cDNA by the cDNA synthesis kit (iNtRON Biotechnology, Seongnam-si, Gyeonggi-do, South Korea) according to the instructions of the manufacturer. The cDNA was amplified using SYBR^®^ Green master mix (Thermo Scientific, MA, USA). The primer sequences are listed in Table S3 (See Supplementary Materials). The 2^−ΔΔCt^ method [45] was followed for the calculation of the relative QS gene expression.

### 3.5. In Vivo Protection Assay

#### 3.5.1. Animals

We obtained thirty male albino mice from the animal house of Cairo University, Egypt. They weighed 22 g to 27 g, and they were allowed to access freely both filtered water and a standard pellet meal. Additionally, they were retained in room temperature and a 12 h light/dark cycle [46]. They were left for one week for acclimation before the beginning of the in vivo study. Our experiment was performed according to the guide of standard laboratory animal care and service. The allocated approval code was TP/RE/08-22P-0031 by the Research Ethical Committee, Faculty of Pharmacy, Tanta University, Egypt.

#### 3.5.2. Experimental Protocol

The survival rate was determined in the different experimental groups to evaluate the ability of the combination to protect the mice against the infection caused by *P. aeruginosa* [9]. In brief, suspensions of *P. aeruginosa* were prepared (1 × 10^8^ CFU/mL) with and without treatment with sub-inhibitory concentrations of the combination. They were intraperitoneally injected into the animals. Then, mice were randomly grouped into three groups (*n* = 10 mice): group I non-infected normal group, group II infected with untreated *P. aeruginosa*, and group III infected with treated *P. aeruginosa*. Survival of the mice was monitored for two weeks.

#### 3.5.3. Statistics

Experiments were conducted in triplicate and the results are presented as mean ± standard deviation (SD). Two-way ANOVA was utilized for evaluating the statistical significance at *p* < 0.05. A Kaplan–Meier survival curve was established for assessing the survival rate of the animals. We used Graph Pad Prism software for the statistical analysis (Version 8, GraphPad Software Inc., San Diego, CA, USA).

### 3.6. Docking Study

The molecular docking study was executed using the new version of Autodock vina 1.2.3 [38,47] as described in Data S1 (See Supplementary Materials).

## 4. Conclusions

In this article, an HPLC-UV study has been developed for comparing the main phytochemical constituents of the different plant parts of two *Pelargonium × hortorum* cultivars with the medicinally used *P. sidoides* root extract. Umckalin was found to be characteristic for *P. sidoides* root extract and absent in all other tested extracts. *P. × hortorum* root extracts can be considered as a rich source for catechin and gallic acid combination and showed the best antibacterial activity against the tested *P. aeruginosa* isolates. Our study revealed that the combination between catechin and gallic acid could be a future medical approach for the treatment of the infections caused by *P. aeruginosa* due to its anti-biofilm and quorum-quenching activities. It was demonstrated that these activities could be attributed to the downregulation of *Las*R and *Las*I genes that are essential for quorum sensing in *P. aeruginosa*, in addition to inhibiting the *Las*R protein as predicted by the simultaneous docking study. More research studies are recommended for confirming the predicted interactions of catechin and gallic acid and the effect of their binding on the activity of *Las*R protein and to illuminate the anti-biofilm potential of this combination against other bacterial species.

## Figures and Tables

**Table 1:** HPLC-UV assay results of detected catechin, gallic acid, scopoletin, and umckalin content at a wavelength of 210 nm using RP-C_18_ column, in *pelargonium sidoides* and two *Pelargonium × hortorum* cultivars; with pink flowers (PPH) and with white-to-rose flowers (WPH).

Samples ^a^	Concentrations of Investigated Biomolecules (μg/mL) ^b^			
	Gallic Acid	Catechin	Scopoletin	Umckalin
*Pelargonium sidoides* root extract	22.00	10.96	0	3.09
PPH root	163.69	172.56	0	0
WPH root	98.75	104.59	0	0
PPH flower	124.99	0	0	0
WPH flower	213.50	0	0	0
PPH leaf	92.29	44.10	0	0
WPH leaf	82.62	47.98	0	0
PPH stem	133.96	32.55	0	0
WPH stem	85.11	37.922	0	0

^a^ Different extracts were analyzed at a concentration of 10 mg/mL. ^b^ The detected concentrations are expressed as the means of triplicate injections.

**Table 2:** Impact of catechin, gallic acid and their combination on the biofilm-forming capability of *P. aeruginosa* isolates.

Biofilm Formation	Optical Density Value	Number of the Isolates			
		Before Treatment	After Treatment with Catechin	After Treatment with Gallic Acid	After Treatment with the Combination
Non-biofilm forming	≥0.124	5	5	6	12
Weak	0.125–0.248	4	5	5	6
Moderate	0.249–0.372	4	4	3	0
Strong	≤0.373	6	5	5	1

**Table 3:** Docking scores of catechin, gallic acid, and their combination against the transcriptional activator receptor (*Las*R) using AutoDock Vina 1.2.3.

Compounds	Binding Energy (kcal/mol)
Catechin and gallic acid simultaneous docking	−13.07
Catechin	−10.29
Gallic acid	−7.059
Co-crystallized compound	−5.352

## Data Availability

Not applicable.

## Supplementary Materials

The following supporting information can be downloaded at: https://www.mdpi.com/article/10.3390/molecules27227841/s1, Figure S1: TLC chromatograms of investigated plant extracts against reference standards; Table S1: Minimum inhibitory concentration (MIC) of investigated extracts; Table S2: Minimum inhibitory concentration (MIC) values of catechin and gallic acid against *P. aeruginosa* isolates; Table S3: Sequences of the utilized primers; Data S1: docking study procedure [38,47,48,49].

## Sample Availability Statement

Samples of the compounds are not available from the authors.
