# Supplementary material for: Anti-Quorum Sensing and Anti-Biofilm Activity of Pelargonium × hortorum Root Extract against Pseudomonas aeruginosa: Combinatorial Effect of Catechin and Gallic Acid
Source: Molecules. 2022 Nov 14;27(22):7841. doi: 10.3390/molecules27227841 (PMC9695561; doi:10.3390/molecules27227841)
Supplement: Supplementary file 1 [file molecules-27-07841-s001.zip › molecules-1998735-supplementary.pdf]

Anti-Quorum Sensing and Anti-Biofilm Activity of *Pelargonium × hortorum* Root Extract against *Pseudomonas aeruginosa*: Combinatorial Effect of Catechin and Gallic Acid

Fatma M. Abdel Bar [1,2,*], Manal A. Alossaimi [3], Engy Elekhnawy [4], May Abdullah Abulrahman Alzeer [1], Amal Abo Kamer [4], Ehssan Moglad [5] and Mai H. ElNaggar [6]

[1] Department of Pharmacognosy, College of Pharmacy, Prince Sattam Bin Abdulaziz University, Al-Kharj 11942, Saudi Arabia
[2] Department of Pharmacognosy, Faculty of Pharmacy, Mansoura University, Mansoura 35516, Egypt
[3] Department of Pharmaceutical Chemistry, College of Pharmacy, Prince Sattam Bin Abdulaziz University, Al-Kharj 11942, Saudi Arabia
[4] Pharmaceutical Microbiology Department, Faculty of Pharmacy, Tanta University, Tanta 31527, Egypt
[5] Department of Pharmaceutics, College of Pharmacy, Prince Sattam bin Abdulaziz University, Al-Kharj 11942, Saudi Arabia
[6] Department of Pharmacognosy, Faculty of Pharmacy, Kafrelsheikh University, Kafrelsheikh 33516, Egypt
* Correspondence: f.abdelbar@psau.edu.sa; Tel.: +966-54540-3617

Table of Contents	Page
**Table S1.** Minimum inhibitory concentration (MIC) values of*pelargonium sidoides* root extract and the methanolic extracts of different parts of two *Pelargonium* × *hortorum* cultivars; with pink flowers (PPH) and with white to rose flowers (WPH) against *P. aeruginosa* isolates.	3
**Table S2.** Minimum inhibitory concentration (MIC) values of catechin and gallic acid against *P. aeruginosa* isolates.	4
**Table S3.** Sequences of the utilized primers.	5
**Figure S1.** TLC chromatograms (Silica gel GF_{254}, using the solvent system $CHCl_3$-CH_3OH, 90:10 v/v) of standards and plants extracts of *Pelargonium sidoides*, *Pelargonium* × *hortorum* cultivars with pink flowers (PPH), and white flowers (WPH) visualized by (a) UV light at 336 nm, (b) UV light at 254 nm, and (c) 10% sulfuric acid spray, including the lanes; (1) PPH flower, (2) PPH leaf, (3) PPH stem, (4) PPH root, (5) WPH flower, (6) WPH leaf, (7) WPH stem, (8) WPH root, (9) Catechin, (10) Gallic acid, (11) Umckalin, (12) Scopoletin, (13) *Pelargonium sidoides*. Black arrows refer to catechin (orange spot).	6
**Data S1.** Docking Study Procedure	7

Table S1. Minimum inhibitory concentration (MIC) values of *pelargonium sidoides* root extract and the methanolic extracts of different parts of two *Pelargonium × hortorum* cultivars; with pink flowers (PPH) and with white to rose flowers (WPH) against *P. aeruginosa* isolates.

**Isolate code**	MIC (µg/mL)								
	***Pelargonium sidoides* root extract**	**PPH root**	**WPH root**	**PPH flower**	**WPH flower**	**PPH leaf**	**WPH leaf**	**PPH stem**	**WPH stem**
**P1**	>2048	512	512	512	1024	>2048	>2048	>2048	>2048
**P2**	1024	512	1024	>2048	>2048	>2048	>2048	>2048	>2048
**P3**	1024	1024	1024	512	1024	1024	1024	1024	1024
**P4**	>2048	1024	1024	512	1024	2048	1024	512	1024
**P5**	>2048	1024	1024	1024	512	>2048	512	>2048	>2048
**P6**	>2048	512	2048	>2048	>2048	>2048	>2048	2048	>2048
**P7**	>2048	512	2048	1024	512	1024	>2048	1024	>2048
**P8**	512	512	512	2048	2048	1024	512	>2048	1024
**P9**	1024	512	512	2048	2048	>2048	1024	>2048	2048
**P10**	1024	2048	512	2048	1024	2048	>2048	>2048	>2048
**P11**	1024	512	1024	1024	1024	>2048	2048	1024	>2048
**P12**	512	512	512	1024	1024	512	2048	1024	2048
**P13**	>2048	1024	1024	1024	1024	>2048	>2048	2048	1024
**P14**	512	1024	1024	1024	2048	1024	2048	2048	2048
**P15**	>2048	1024	1024	1024	2048	1024	2048	>2048	>2048
**P16**	2048	512	1024	2048	2048	2048	1024	>2048	>2048
**P17**	2048	512	2048	>2048	>2048	>2048	>2048	>2048	>2048
**P18**	>2048	512	2048	>2048	>2048	>2048	>2048	1024	2048
**P19**	2048	512	2048	2048	512	>2048	1024	512	1024
**P reference**	512	512	1024	512	1024	1024	1024	1024	1024

Table S2. Minimum inhibitory concentration (MIC) values of catechin and gallic acid against *P. aeruginosa* isolates.

Isolate code	MIC (µg/mL)			Isolate code	MIC (µg/mL)		
	Ciprofloxacin	Catechin	Gallic acid		Ciprofloxacin	Catechin	Gallic acid
P1	16	64	128	P11	128	64	128
P2	32	1024	512	P12	16	1024	512
P3	32	1024	128	P13	16	512	64
P4	16	64	256	P14	32	128	128
P5	32	256	128	P15	32	512	128
P6	32	512	1024	P16	16	64	1024
P7	32	128	512	P17	32	512	256
P8	16	64	512	P18	256	512	1024
P9	16	1024	1024	P19	128	1024	256
P10	256	512	128	P reference	0.5	16	32

Table S3. Sequences of the utilized primers.

Gene name	Primer sequence
*opr*L	F 5`-AACAGCGGTGCCGTTGAC-3`<br>R 5`-GTCGGAGCTGTCGTACTCGAA-3`
*las*I	F 5`-CGCACATCTGGGAACTCA-3`<br>R 5`-CGGCACGGATCATCATCT-3`
*las*R	F 5`-CTGTGGATGCTCAAGGACTAC-3`<br>R 5`-AACTGGTCTTGCCGATGG-3`

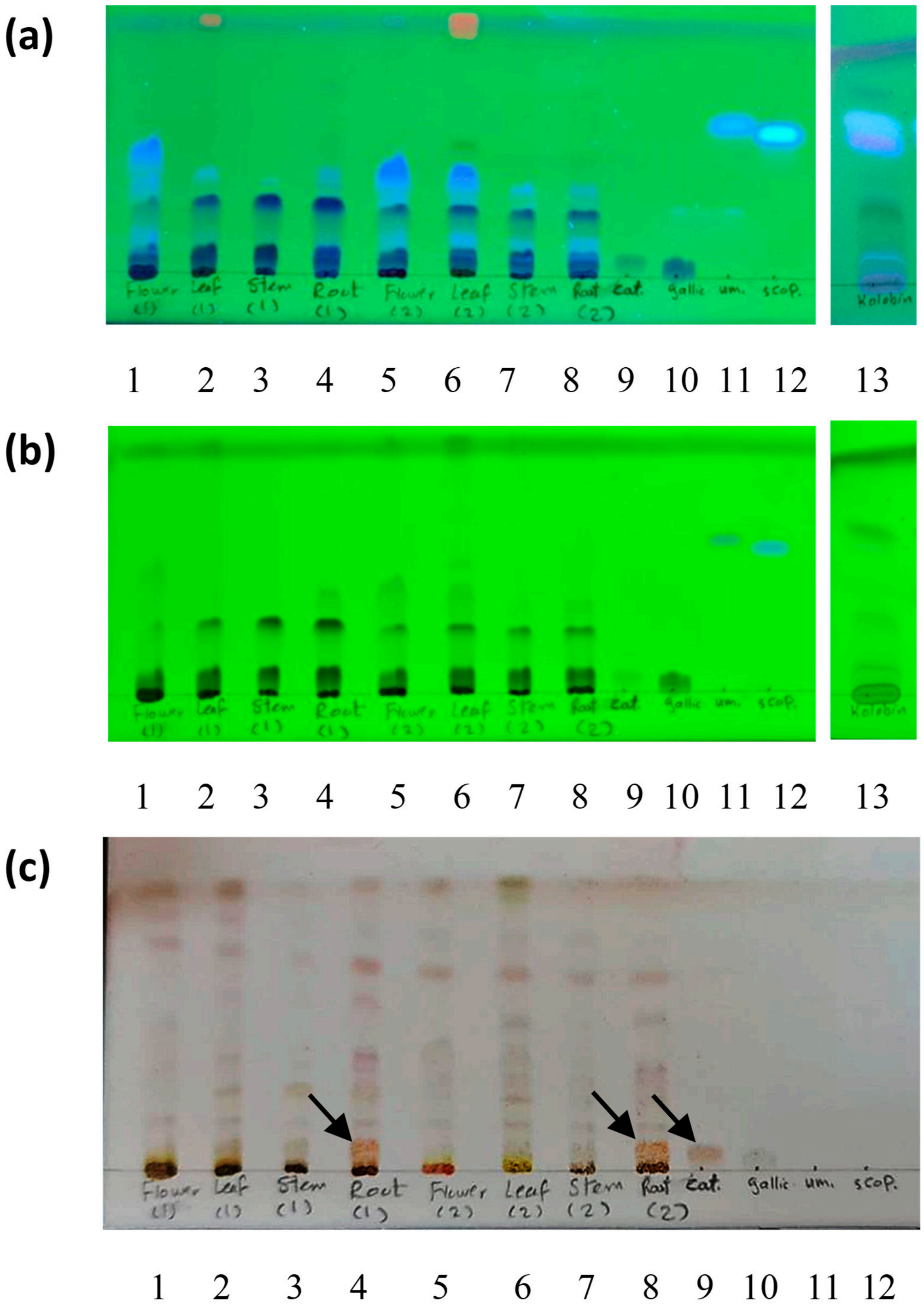

Figure S1. TLC chromatograms (Silica gel GF$_{254}$, using the solvent system $CHCl_3$-CH_3OH, 90:10 v/v) of standards and plants extracts of *Pelargonium sidoides*, *Pelargonium* × *hortorum* cultivars with pink flowers (PPH), and white flowers (WPH) visualized by (a) UV light at 336 nm, (b) UV light at 254 nm, and (c) 10% sulfuric acid spray, including the lanes; (1) PPH flower, (2) PPH leaf, (3) PPH stem, (4) PPH root, (5) WPH flower, (6) WPH leaf, (7) WPH stem, (8) WPH root, (9) Catechin, (10) Gallic acid, (11) Umckalin, (12) Scopoletin, (13) *Pelargonium sidoides*. Black arrows refer to catechin (orange spot).

Data S1. Docking Study Procedure

The molecular docking study was executed using the new version of Autodock vina 1.2.3 [38, 47]. The crystal structures for the transcriptional activator receptor (*Las*R, PDB code: 2UVO) were downloaded from the RCSB protein data bank in PDB format. The structures of catechin and gallic acid were drawn using ChemDraw and converted to PDB formats using Pymol software [48]. The investigated compounds and the used proteins crystal structures were prepared for the docking study using Autodock tools. *Las*R binding site coordinates were determined using a grid box around the co-crystallized autoinducer, 3-oxoC12-acylhomoserine lactone [49]. The binding site was validated by redocking of the co-crystallized autoinducer. A grid box with the dimensions of 30 × 30 × 30 and spacing of 0.375 Å with X, Y, and Z coordinates of 23.633, 16.819, 80.434 was used. The obtained docking poses of catechin and gallic acid with the least RMSD values were visualized using Pymol [48].
